# Correlation of frontal prism structures and slope failures near the trench axis with shallow megathrust slip at the Japan Trench

**DOI:** 10.1038/s41598-020-68449-6

**Published:** 2020-07-14

**Authors:** Yasuyuki Nakamura, Toshiya Fujiwara, Shuichi Kodaira, Seiichi Miura, Koichiro Obana

**Affiliations:** 1 Japan Agency for Marine-Earth Science and Technology (JAMSTEC), 3173-25 Showa-machi, Kanazawa-ku, Yokohama, Kanagawa 236-0001 Japan; 2 Japan Agency for Marine-Earth Science and Technology (JAMSTEC), 2-15 Natsushima-cho, Yokosuka, Kanagawa 237-0061 Japan

**Keywords:** Natural hazards, Solid Earth sciences

## Abstract

Since the giant 2011 Tohoku earthquake and tsunami, much research has focused on the distribution of coseismic slip at shallow depths during this subduction megathrust event. Here we present seismic images obtained in the immediate vicinity of the trench axis, that show thrust faults and fold-and-thrust type deformation structures near the epicenter of the 2011 Tohoku earthquake where the large coseismic slip has been inferred, and chaotic structure and the absence of thrust faults in northern and southern source areas. Seismic profiles show evidence of slope failures of the trench inner wall in a proposed tsunami source region around 39°–40° N, where the slips estimated from previous studies are in disagreement. Our results show that structural characteristics in the trench axis may be related to the occurrence of shallow megathrust slip and tsunamigenesis in the Japan Trench.

## Introduction

**Figure 1:**
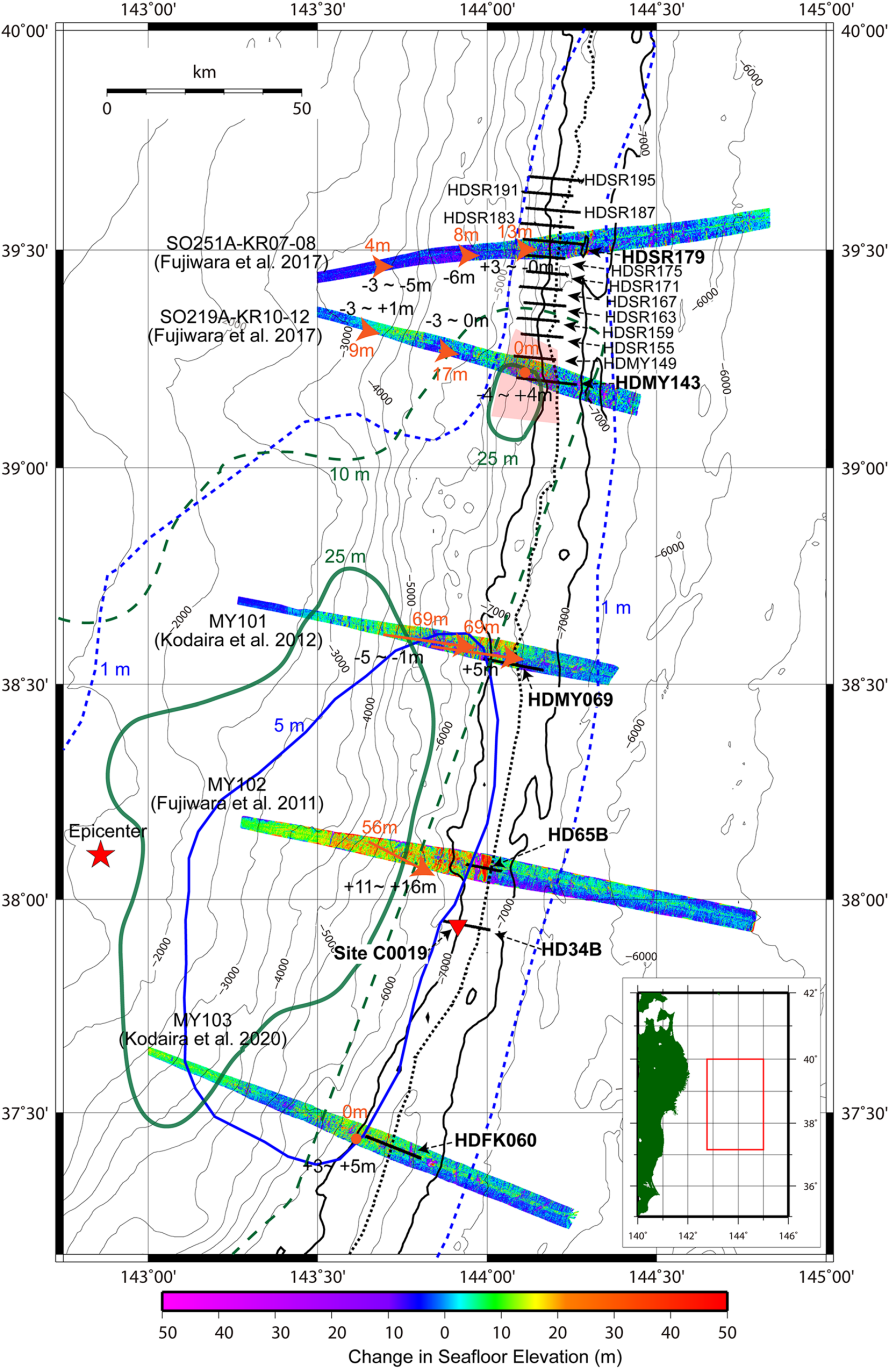
Changes in seafloor elevation after the 2011 Tohoku earthquake and locations of seismic lines. Bathymetric contours are in meters; the 7,000 m contour delineates the trench axis. Thick black dotted line corresponds to the toe of the landward slope. The map location is indicated by the red rectangle in the inset map. Orange arrows and numbers indicate trench-normal displacement, and black numbers indicate vertical displacement, estimated by using bathymetric data from before and after the Tohoku earthquake^8–11^. Bold black lines indicate the locations of the seismic profiles shown in Figs. 2 and 3. Green contours outline the area of large slip (solid contour, ≥ 25 m; dashed contour, ≥ 10 m) of the 2011 Tohoku earthquake obtained by tsunami inversion^6^, and blue contours indicate the area of sea surface elevation (solid contour, ≥ 5 m; dashed contour, ≥ 1 m) determined by tsunami inversion^5^. The red shaded polygon indicates the tsunami source region, estimated from ocean-bottom electromagnetometer data^23^. The map and arrows of changes in seafloor elevation were reproduced from the data published in ref.^11^. The bathymetry data JTOPO30v2 by Marine Information Research Center, Japan Hydrographic Association was used to draw the contour in this figure. The map was created with GMT-4.5.7 (https://www.generic-mapping-tools.org/)^35^.

The giant (M9) 2011 Tohoku earthquake ruptured the plate boundary fault in the Japan Trench subduction zone through the shallowest part of the megathrust, and this shallow slip in particular enhanced the destructive tsunami that followed. Numerous rupture models have been provided to elucidate the characteristics of this giant earthquake using seismological^1,2^, geodetic^3^, and tsunami^4,5^ data, and combinations of these^6^. One distinctive feature is a large (~ 50 m) shallow megathrust slip that occurred at around 38°–38°30′N. Another characteristic is a proposed tsunami source region at around 39°–40°N, to the north of the large shallow slip area identified by tsunami data analyses^4–6^. Seafloor geodetic observations^7^ and estimates of seafloor displacement based on bathymetric datasets^8–11^ are strong evidence with which to examine the propagation of fault slip towards the seafloor through the trench axis area. Research using differential bathymetry has shown that seafloor displacement during the Tohoku earthquake was > 50 m near the trench axis at 38°05′–38°35′N, where the large slip occurred during the Tohoku earthquake^8,9^, but was not significant outside of this area^10,11^ (Fig. 1). Interestingly, the displacement estimated from differential bathymetry was not significant near the trench axis at 39°10′–39°30′N^10^, the area proposed as a northern source region of the tsunami^4–6^. The structure of deformation within the overriding plate at the toe of the frontal prism near the epicentre is considered an important characteristic for understanding shallow megathrust events such as the Tohoku earthquake^9^.

Here we present seismic profiles acquired at the immediate vicinity of the Japan trench axis, including those obtained at the same locations as the differential bathymetry estimates. We use these results in combination with seismic profiles in the area near the proposed shallow slip and tsunami source to examine the seismic structure in the vicinity of the trench axis. We find that the structural characteristics seen in the seismic profiles are related to seafloor displacement and possible tsunami generation.

## Results

### Seismic profiles in the vicinity of the differential bathymetry estimates

**Figure 2:**
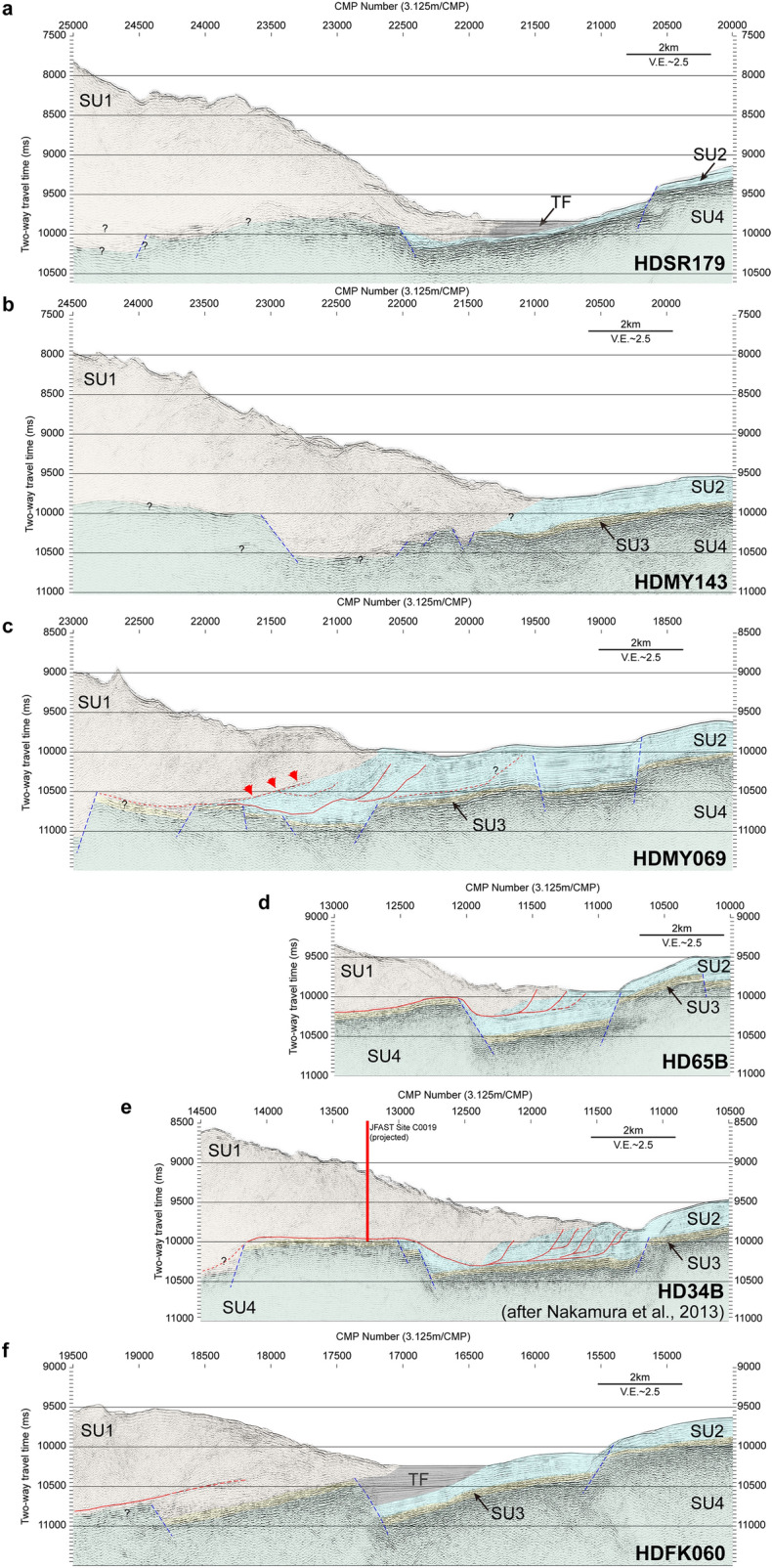
Seismic profiles with interpretation. Post-stack time-migrated sections, with interpretation shown by colour, along seismic survey lines (**a**) HDSR179, (**b**) HDMY143, (**c**) HDMY069, (**d**) HD65B, (**e**) HD34B^12^, and (**f**) HDFK060 (locations shown in Fig. 1), which correspond to the locations of differential bathymetry estimates. Seismic units SU1 (brown), SU2 (blue), SU3 (yellow), SU4 (green), and TF (grey) are described in the text. Dashed lines indicate normal faults (blue) and thrust faults (red). Red arrows in Fig. 2c indicate a low-angle dipping reflection interpreted as a thrust fault. Vertical exaggeration is 2.5 ×, assuming a seismic velocity of 1,500 m/s. The seismic profile of HD34B was reproduced from the data published in Ref.^12^.

The time-migrated seismic profiles clearly reveal detailed structures in the vicinity of the Japan Trench axis, including incoming sediment on the Pacific plate, bending-related normal faults, the oceanic basement, and reverse faults and fold structure within the vicinity of the trench axis. We interpreted these seismic sections in terms of four “seismic units” (SU) following a previous study^12^, as shown in Fig. 2a–f: SU1 is interpreted as acoustically chaotic frontal prism sediment, SU2 as incoming hemipelagic and pelagic soft sediment, SU3 as chert, and SU4 as igneous basement. We additionally interpreted a stratified unit TF, which shows a clear onlap relationship with SU2 at the trench axis, corresponding to the trench fill sediments (Fig. 2a,f). Three of the seismic profiles in Fig. 2c–e were acquired in the area with the huge shallow slip during the 2011 Tohoku earthquake around 38°–38°30′N. Seismic profiles HDMY069 and HD65B (Fig. 2c,d), where differential bathymetry suggested a large horizontal displacement^8,9^, show deformation of the sediment units by thrust faults in the vicinity of the trench axis. Seismic profile HD34B (Fig. 2e), located very close to the JFAST drill site C0019, where the coseismic slip was suggested to reach near the trench axis^13–15^, shows similar deformation^12^. Three other seismic profiles (Fig. 2a,b,f), located outside of the area with very large shallow slip, display a chaotic acoustic character and no clear thrust faults in the vicinity of the trench axis. The differential bathymetry indicated little or no trench-normal horizontal displacement along these profiles^10,11^.

### Seismic profiles in the northern tsunami source area

**Figure 3:**
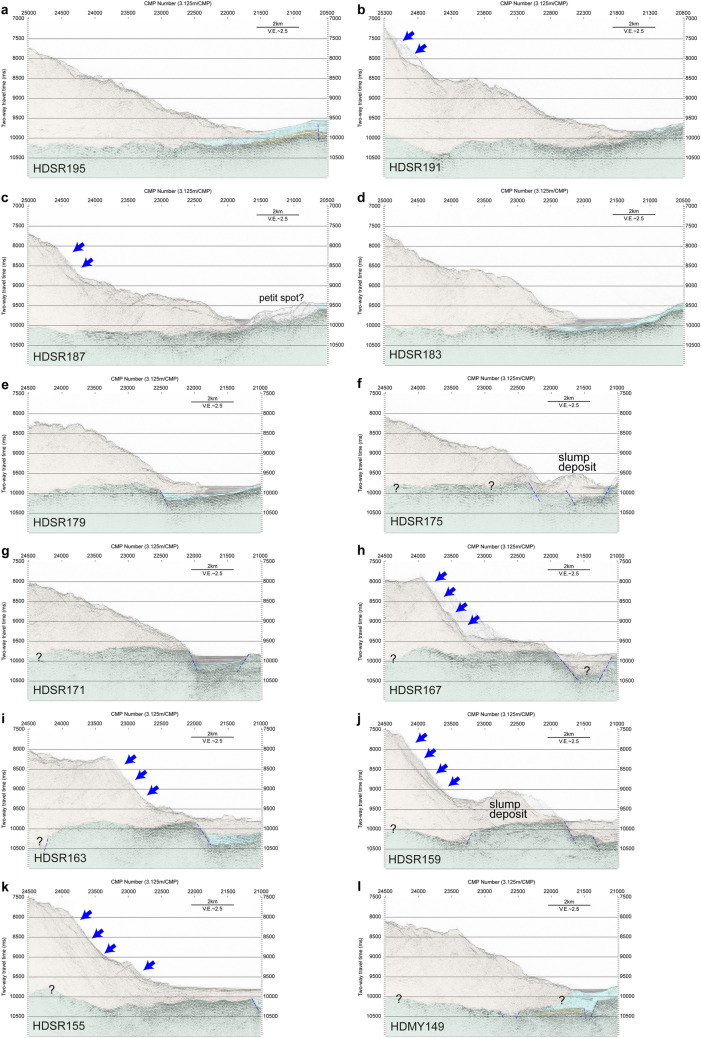
Seismic profiles around 39°30′N. Post-stack time-migrated sections with interpretation shown by colour, along seismic lines (**a**) HDSR195, (**b**) HDSR191, (**c**) HDSR187, (**d**) HDSR183, (**e**) HDSR179, (**f**) HDSR175, (**g**) HDSR171, (**h**) HDSR167, (**i**) HDSR163, (**j**) HDSR159, (**k**) HDSR155, and (**l**) HDMY149. Interpreted seismic units are the same as Fig. 2. Profile locations are shown in Fig. 1. Blue arrows indicate steep slopes. Vertical exaggeration is 2.5 ×, assuming a seismic velocity of 1,500 m/s.

Figure 3a-l shows 12 seismic profiles of the trench axis and lowermost landward slope between around 39°12′N and 39°40′N, an area that includes the proposed northern tsunami source region^4–6^ (see Fig. 1). Although some profiles include sideswipe reflections, in general, all of these profiles show an acoustically chaotic structure and no thrust faults within the sediment near the trench axis. Six profiles (HDSR191, HDSR187, HDSR167, HDSR163, HDSR159, and HDSR155) depict both steep slopes (blue arrows in Fig. 3b,c,h–k) and possible slope failures in the trench inner wall, whereas the other profiles, although they do not show steep slopes, also suggest slope failures. Several seismic profiles, especially lines HDSR175 and HDSR159, also show slump deposits overlying the incoming sediments in the trench axis.

## Discussion

### Shallow megathrust slip and seismic image in the trench axis

Published rupture models of the 2011 Tohoku earthquake proposed by seismological^1,2^, geodetic^3^, and tsunami^4–6^ inversions largely agree in placing the largest slip at around 38°–38°30′N. Seismic profile HDMY069 and HD65B^9^ (Fig. 2c,d), located at 38°35′N and 38°05′N respectively, displays fold-and-thrust structures imaged within the sediment in the trench axis, where differential bathymetry suggested a large (~ 50–70 m) trenchward displacement during the Tohoku earthquake^8,9^. Similarly, folding and thrust faults have been identified near the trench axis at around 37°55′N (HD34B, Fig. 2e)^12^, very close to the JFAST drill site C0019, where borehole temperature measurements strongly suggest that the fault rupture during the Tohoku earthquake nearly reached the trench axis^15^. However, other seismic profiles (Fig. 2a,b,f), obtained outside of the largest shallow slip area, display an acoustically chaotic structure and no clear folds or thrust faults in the trench axis. The differential bathymetry suggested no significant trench-normal horizontal displacement during the Tohoku earthquake in the vicinity of these three profiles. One possible interpretation of these observations is that thrust faults are present within the trench axis area at 37°55′–38°35′N, but absent at 37°25′N and 39°12′–39°40′N. An alternative interpretation, however, is that thrust faults, although present, were not clearly imaged by the seismic profiles obtained at 37°25′N and 39°12′–39°40′N. Possible reasons why the faults might not be clearly imaged in the Japan Trench are that (1) as a result of erosion the original sediment strata are not preserved, so that the faults, even if they exist, cannot be clearly distinguished by the offset of strata or that (2) the acoustic impedance contrast between the faults or fault zone and the surrounding area is not strong enough to be imaged by the seismic method used. At site C0019, existence of a fault zone is suggested within the hanging-wall sediments above the plate boundary fault by logging data analysis, but they are not clearly imaged on the seismic profile crossing the site^16^. Seismic sections in Fig. 2 except for the Line HDMY143 (Fig. 2a) show all the seismic units in the incoming plate (SU2–SU4), which may indicate that the incoming sediments were not largely eroded in the most of the Japan Trench axis.

Seismic profiles along Lines HDMY069, HD65B, and HD34B clearly depict thrust faults as having displaced sediment strata and caused related folding deformation (Fig. 2c–e). Because thrust faults are observed along the differential bathymetry profiles showing large coseismic seafloor displacement (Fig. 2c–e), the imaged thrust faults can be interpreted as the shallowest portions of the plate boundary fault in the trench axis. The seismic profile of Line HDMY069 also depicts a low-angle reflection, interpreted as a thrust fault, within the acoustically chaotic sediment layer (red arrows in Fig. 2c). The existence of this reflection suggests a well-developed thrust fault or fault zone that has a strong enough acoustic impedance contrast to allow it to be imaged against the surrounding chaotic sediments. The high amplitude reflections from strong acoustic impedance contrast may be related to the high pore pressure associated with the fault zone as inferred in the central America^17,18^ and the Nankai Trough^19,20^ subduction zones. We suggest that the thrust faults clearly imaged in the trench axis acted as the shallowest plate boundary megathrust during the Tohoku earthquake, and caused the large shallow slip proposed by seismological studies and seafloor displacement observed by the differential bathymetry. The high pore pressure associated with the fault zone might promote the shallow slip. We have to note that the area discussed in this study is narrower than the horizontal resolution of the slip models proposed from seismological, geodetic, and tsunami data analyses. The megathrust slip which reached near the trench but not to the seafloor could cause the deformation of the hanging wall prism^21^. If the coseismic slip reached to the seafloor and the hanging wall moved as a rigid block, it might not cause the deformation within the hanging wall. When combining the results from different methods with different resolutions, careful consideration of those differences would be necessary to understand the shallow slip behavior of the subduction megathrusts. Based on the correlation between the structures observed in the seismic sections and the distribution of the slip in the 2011 Tohoku earthquake, we speculate that the imaged thrust faults and folding structures in the vicinity of the trench axis may be a proxy of past slip to the trench^11^.

### Slope failure observed in the northern tsunami source region of the 2011 Tohoku earthquake

**Figure 4:**
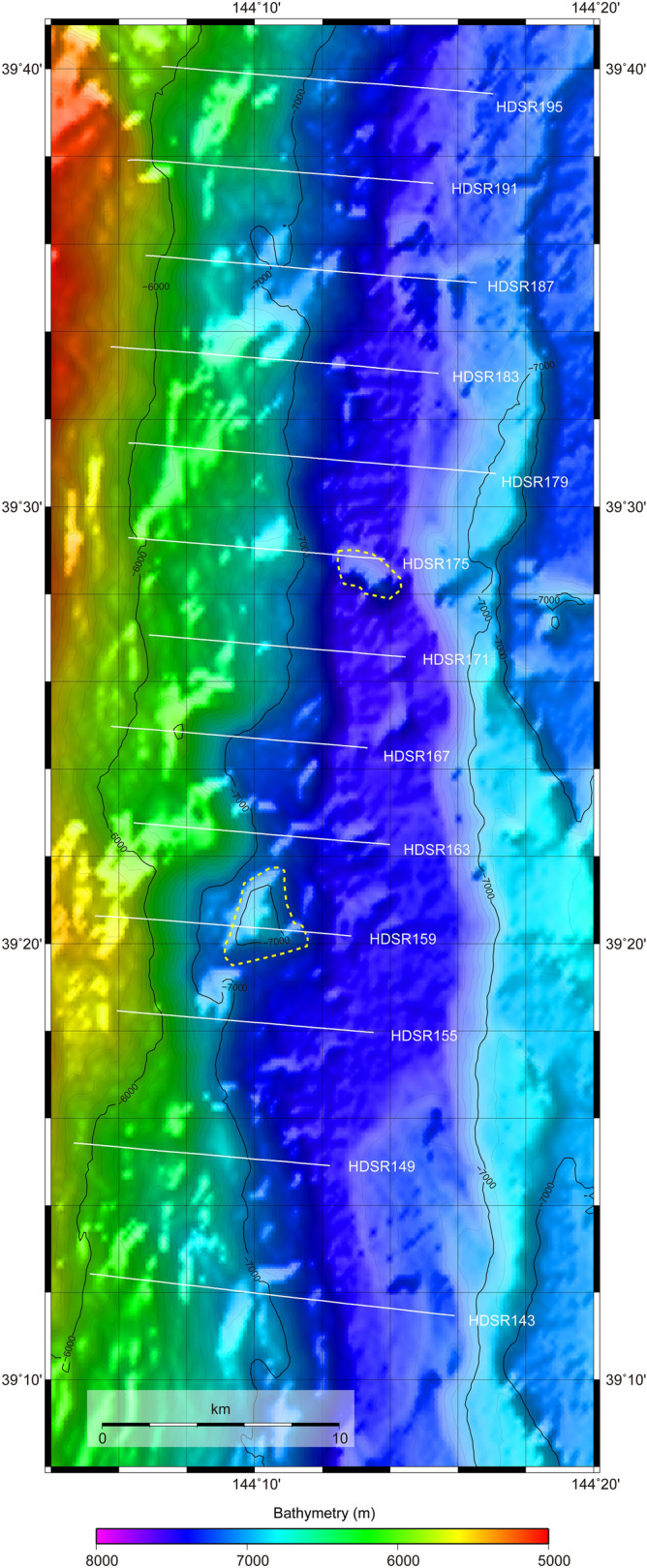
Bathymetry around 39°30′N. Shaded bathymetric map generated from compiled pre-earthquake bathymetric data^27^. Locations of the seismic sections shown in Fig. 3 are also indicated. Yellow dashed lines delineate slump bodies that were also identified on the corresponding seismic sections. The map was created with GMT-4.5.7 (https://www.generic-mapping-tools.org/^35^.

The seismic profiles clustered around 39°30′N (Fig. 3a-l) suggest that pervasive slope failure has occurred in the past in this area, which extends at least 40 km along the trench. Seismic sections of lines HDSR159 and HDSR175 in particular show possible slump deposits (Fig. 3f,j) with a horizontal size of ~ 3 km (Fig. 4) that presumably represent a very recent geological process because they overlie the incoming sediments in the trench. Slumping and slope failure are potentially tsunamigenic^22^, and a tsunami source near this region has been proposed on the basis of tsunami data analyses from the Tohoku earthquake^4–6^. A tsunami source in the lower landward slope at around 39°10′N–39°15′N has also been inferred by an analysis of ocean-bottom electromagnetometer data^23^ (Fig. 1). A submarine mass failure (SMF) around 39°30′N was proposed as an additional tsunami source to accommodate the high amplitude tsunami waveforms off Iwate, north of the epicentre^24^. However, based on a detailed review of various studies on the rupture process of the Tohoku earthquake, Lay^25^ concluded that “an exotic source such as a slump or inelastic deformation is not required to account for the runup (although that possibility cannot be ruled out)”. The amount of the slip along the plate boundary faults has been estimated as ~ 35 m^4^, ~ 25 m^6^, or 30–40 m^26^ around 39°30′N by tsunami data analysis. On the other hand, the differential bathymetry indicates that the trenchward horizontal displacement of the lowermost landward slope during the Tohoku earthquake, if any, did not exceed the uncertainty of the estimates. The estimated value was ~ 29 m at maximum around 39°12′N^10^, which is comparable to or slightly smaller than the values estimated from tsunami data. Differential bathymetry estimates suggested that the existence of localized very large slip of the megathrust in the shallow part near the trench is unlikely to explain the tsunami source, and the estimated direction of the seafloor shift was mainly trench-parallel and the trench-normal shift was small^10^. This predominantly trench-parallel shift might have been caused by the broader small uplift in the lower slope^10^. It is also possible that bathymetric features such as small bumps or valleys caused by slope failures obscured the correlation between the bathymetric data from before and after the earthquake. Taking into account the slope failures imaged in our seismic profiles of this region, we speculate that small-scale slope failure might have contributed to tsunamigenesis during the Tohoku earthquake. Note that no large-scale slope failure associated with the 2011 Tohoku earthquake was identified in the differential bathymetry result in this region, including the area of the proposed SMF^10,24^. The slope failures observed in this study is located ~ 20 km trenchward of the area of the proposed SMF^24^.

Bathymetric data obtained before the 2011 Tohoku earthquake show the presence of slump deposits in this area^27^ (Fig. 4), so slope failure undoubtedly occurred before the Tohoku earthquake. The acoustically chaotic character of the inner trench hanging wall on some of the seismic sections might be due to repeated slope failures and erosion. The slump deposits observed on the seismic sections and in the bathymetry data were not, at least initially, formed during the Tohoku earthquake, but they might be related to past tsunamis. This area has been suggested to be the source region of the 1896 Meiji Sanriku earthquake and tsunami^21,28^. Although the estimated large slip (~ 20 m) region is landward of our survey area^29^, the observed slump deposit might be related to the 1896 Meiji Sanriku event.

The structure and property of the incoming plate are important controlling factors of the seismogenesis and structural evolution in the subduction zones. A recent study^30^ found that the post-spreading volcanism altered the structure of the upper most part of the incoming Pacific plate in the vicinity of the trench axis around 39°N, which corresponds to the area where slope failures were identified in the seismic profiles. Magmatic intrusions and thermal metamorphism associated with post-spreading volcanism were suggested to disturb the smectite-rich pelagic clay layer in incoming sediments, and the subduction of this disturbed area was suggested to prevent giant near-trench interplate coseismic slip in this region^30^. Most of the seismic profiles around 39°30′N were interpreted to lack the chert layer (SU3) and suggest that the pelagic clay layer was disturbed in the shallow most part of the subduction zone. Seismic profiles (e.g., Fig. 3g–j) show rough basement topography and a small hill-like structure interpreted as post-spreading volcanism (petit-spot, Line HDSR187, Fig. 3c). The absence of the smectite-rich clay around the plate boundary fault could have increased the basal friction^30^. The increased basal friction could have contributed to building higher critical taper angle and steeper slopes of the frontal prism^31^. The taper angle estimated from our seismic sections in the small shallow slip area (Fig. 2a,b,f) is approximately 60% higher than that in the large shallow slip area (Fig. 2c–e) on average, assuming 1.8–2.1 km/s as the P wave velocity within the hanging wall sediments^32,33^. The subduction of the basement with rough topography might have also caused the steep inner trench slopes. The steeper slopes could have caused slope failures observed in this region. The taper angles in the Japan Trench were also estimated using regional-scale seismic profiles^34^. The area of seamount subduction has a larger taper angle^34^, which is in good agreement with our study.

## Summary

Various studies have shown that the large shallow plate boundary slip during the Tohoku earthquake occurred in the central part of the Japan Trench but did not occur in other parts. Seismic profiles near the Japan Trench axis acquired in the vicinity of the differential bathymetry estimates show that the difference in the amount of the shallow slip during the earthquake corresponds to different characteristic structures in the trench axis. Profiles in areas with large slip display folds and thrust faults, whereas ones in areas without large slip display chaotic acoustic structures in the trench axis without any clearly imaged thrust faults or folding deformation. The deformation structures created by thrust faults in the trench axis may thus be related to large shallow slip of the plate boundary fault during the 2011 Tohoku earthquake. The faults moved the hanging wall, and its displacement was observed in the differential bathymetry data. In the area near a proposed tsunami source at around 39°–40° N, seismic profiles and bathymetry data show slump deposits, suggesting that slope failures have occurred in the past. Considering the slip estimation from tsunami and differential bathymetry data, we speculated that small-scale slope failure might have contributed to tsunamigenesis during the 2011 Tohoku earthquake. Observations in the northern tsunami source area could be related to the subduction of incoming plate altered by post-spreading volcanism, which could increase the basal friction of the frontal prism caused by the absence of the basal input sediments.

## Methods

The seismic dataset used in this study was collected during cruise KR13-11 of R/V *Kairei* in 2013 and cruise KY15-14 of R/V *Kaiyo* in 2015. A digital streamer cable with 168–192 channel hydrophones at 6.25 m intervals, towed at 6 m depth, recorded seismic signals from an array of airguns (6.23 L) towed at 5 m depth. The data were sampled at 1 ms intervals, and airguns were fired every 37.5 m with 13.8 MPa of air pressure. We obtained the best time-migrated images from the recorded data by using conventional procedures described in Ref.^12^.

## Data Availability

The seismic data used in this paper are available from the Japan Agency for Marine-Earth Science and Technology (http://www.jamstec.go.jp/obsmcs_db/e/).
